# A proposed systems approach to the evaluation of integrated palliative care

**DOI:** 10.1186/1472-684X-9-8

**Published:** 2010-05-10

**Authors:** Daryl Bainbridge, Kevin Brazil, Paul Krueger, Jenny Ploeg, Alan Taniguchi

**Affiliations:** 1 Department of Clinical Epidemiology and Biostatistics, McMaster University, Juravinski Cancer Centre, 699 Concession St. Rm 4-203, Hamilton, ON L8V 5C2 Canada; 2 Department of Clinical Epidemiology and Biostatistics, McMaster University, Division of Palliative Care, Department of Family Medicine, McMaster University, St. Joseph's Health System Research Network, 105 Main Street East, Level P1, Hamilton, ON L8N 1G6 Canada; 3 Department of Family and Community Medicine, University of Toronto, 263 McCaul Street, Room 325, Toronto, ON M5T 1W7 Canada; 4 School of Nursing, McMaster University, Department of Health, Aging and Society, McMaster University, Health Sciences Centre, 1200 Main St. W., Room 3N28G, Hamilton ON L8N 3Z5 Canada; 5 Division of Palliative Care, Department of Family Medicine, McMaster University, Health Sciences Centre, 1200 Main St. W., Room 4X22, Hamilton ON L8N 3Z5 Canada

## Abstract

**Background:**

There is increasing global interest in regional palliative care networks (PCN) to integrate care, creating systems that are more cost-effective and responsive in multi-agency settings. Networks are particularly relevant where different professional skill sets are required to serve the broad spectrum of end-of-life needs. We propose a comprehensive framework for evaluating PCNs, focusing on the nature and extent of inter-professional collaboration, community readiness, and client-centred care.

**Methods:**

In the absence of an overarching structure for examining PCNs, a framework was developed based on previous models of health system evaluation, explicit theory, and the research literature relevant to PCN functioning. This research evidence was used to substantiate the choice of model factors.

**Results:**

The proposed framework takes a systems approach with system structure, process of care, and patient outcomes levels of consideration. Each factor represented makes an independent contribution to the description and assessment of the network.

**Conclusions:**

Realizing palliative patients' needs for complex packages of treatment and social support, in a seamless, cost-effective manner, are major drivers of the impetus for network-integrated care. The framework proposed is a first step to guide evaluation to inform the development of appropriate strategies to further promote collaboration within the PCN and, ultimately, optimal palliative care that meets patients' needs and expectations.

## Background

Palliative care, support to help those at end-of-life spend their remaining time in comfort and dignity, has evolved over time with better understanding of the complex needs of those living with advanced illness and with growing acknowledgement of the importance of this health issue [1,2]. Palliative care services can be provided in the home, hospitals, long-term care facilities, and hospices; ideally, within the context of ongoing assessment and management of the multiple physical, psychosocial, and spiritual facets of need [3]. While not all dying people require or desire the same types of professional palliative care services [4,5], requests for interventions to alleviate both symptom distress and family caregiver burden are common in the last year of life [6,7].

Providing the necessary complement of professional services to palliative care patients and their families in the community is a challenge in the current health care environment. The aging population and the changing epidemiology of serious chronic disease, coupled with the mounting costs of institutionalization [8] are straining health care systems [9,10]. Community-based health services, such as those for palliative care in the home, are often highly fragmented due to a combination of diverse professional groups, organizations, and approaches to care [11,12]. A considerable body of evidence shows the prevalent under-identification of those in the palliative stage of illness who have significant distress (including pain and psychosocial conditions). It has been estimated that 60 to 80% of this population remains untreated for these concerns [6,13].

Realizing dying peoples' needs for complex regimens of treatment and social support in a seamless, fiscally responsible manner, and the difficulty of organizing these services in the community are major drivers of the impetus for multi-level strategies to better coordinate palliative care. This has fuelled global interest in integrated service delivery, involving the implementation of collaborative, responsive, cost-effective systems of care at the local level [14-16]. In many counties such as Canada, Netherlands, Australia, and the UK, these integrated systems of care have been mandated by formal policy initiatives in the form of regional palliative care networks [5,14,15,17].

According to authoritative sources on integrated delivery systems of care, namely Provan [12] and Shortell [18], these systems are defined by networks of health care organizations and professionals who work together to coordinate services to meet their patients' needs. Conceptually, these networks are a way of linking fragmented services by increasing inter-organization interactions and ultimately maximizing system efficiency and seamlessness of patient transition [19,11]. The integration of activities between network agencies can include shared staff, joint policy development, joint training programs or workshops, and shared information.

Relative to palliative care, service networks often germinate from informal arrangements between health care providers dedicated to serving the end-of-life needs of those living in their communities. For the purposes of this paper, we define a *formalized *network as a more evolved, organized system of care, as should be evident in the advent of government mandated structuring. At a minimum, this would represent a membership-based group with multi-disciplinary representation from a variety of care settings. This network would be overseen by an executive board or steering committee, enacted to provide leadership and direction in the local provision of palliative care services. Optimally, these palliative care systems include members with decision making and resource allocating authority, and representation from the community, academia, and healthcare institutions, operating with policies and information systems that are shared among providers within the network.

Although community-based network and collaborative palliative care team objectives have been delineated in the literature, there remains little explicit direction on how to operationalize these goals or how such initiatives should be evaluated [6,20,21]. Formalized health care networks, even with key structures such as resources and policy in place, sometimes fail to achieve the goal of integration and ultimately, improvements in patient care. This is largely due to system barriers and poor interdependent functioning among members, left unexamined [22,23]. Superficial evaluations that rely on anecdotal or service use information may be adequate for informing network development in the early formative stages. However, once these networks become more formalized with top-down involvement, this growing complexity necessitates a more comprehensive evaluative approach to competently identify system gaps.

With the increasing prominence of integrated service models in palliative care, and the precarious nature of these arrangements, there is a need for a comprehensive conceptual framework to better understand the structure, process, and outcome functioning of these systems of care. While models of community disease management, such as Wagner's Chronic Care Model [24] have been proposed, these are offered more broadly as compendiums of principle components of a system of service, rather than as an evaluation framework. Furthermore, many of these disease care models have self-management and wellness orientations, not suited to a palliative care application. In the absence of an appropriate model to guide inquiry, the purpose of this paper is to offer, as a starting point, a multi-level evaluative framework for examining palliative care networks (PCNs) using a systems approach.

This paper begins with a presentation of our proposed framework and a general outline of the framework development process. This is followed by an introduction to the three-tier systems approach taken in this framework and an overview of the principal theoretical constructs included. Finally, the individual factors in the framework are described within each system level considered.

### Framework Development

**Figure 1:**
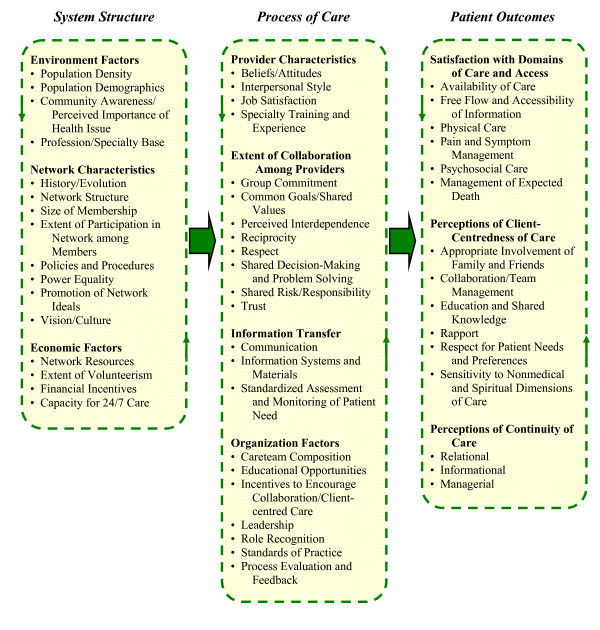
**Conceptual framework for the evaluation of integrated palliative care networks**.

A conceptual framework explains the main things to be studied - the key factors, constructs, or variables - and the presumed relationships among them [25]. The framework we propose for examining palliative care system network functioning is presented in Figure 1. This framework focuses on describing the nature and extent of inter-professional collaboration, which is the central constituent of PCNs, but also takes into account features of the prevailing health care environment. This framework is based on existing models and principles of health system evaluation, explicit theory, consideration of the empirical literature on determinants and indicators of inter-professional collaboration, and previous evaluations of palliative care systems. Constructs related to network success and sustainability, such as community readiness and client-centred care, have also been integrated into the proposed framework.

This framework represents an amalgamation of empirically-supported criteria, with each element making an independent contribution to the description and assessment of the network. Examining each element in the framework is of diagnostic value in that it can specifically direct where intervention is required to improve the overall system. Key features of health system evaluation reflected in the conceptual framework include the use of theory-driven variables and a multi-tiered, systems approach. A theory-driven approach was employed to select potential predictor variables that were associated with the constructs of interest because atheoretical studies are prone to excluding potentially important factors [25,26]. In addition, the use of a theoretical framework to drive the research plan provides for a more systematic, valid, and empirically-sound method of study design, instrument development or selection, and analysis.

### A Systems Approach

The proposed framework is divided into *System Structure*, *Processes of Care*, and *Patient Outcome *(SPO) levels of consideration originally represented in Donabedian's S-P-O model [27]. This systems approach is frequently cited as a requirement of research on healthcare quality [28,29], and is particularly relevant to the examination of community partnerships [30]. In a health care context, 'Structure' is the availability of material and human resources, as well as, organizational characteristics and the physical, social, and economic environment present. 'Process' refers to activities and transactions that constitute health care, usually carried out by professional personnel (but also by non-professionals). Finally, 'Outcome' is the change in individuals attributable to the care they receive. These three tiers are inextricably linked in that system structure contributes to processes, which in turn influence patient outcomes.

Evaluative research in palliative care provision typically focuses on either patient outcomes or provider perceptions of the process. To understand the underlying mechanisms to PCN functioning and in order to be able to make informed recommendations, requires a systems approach where the antecedents to processes and, in turn, patient outcomes are delineated. While the process level attributes may best capture network operations, structure features also need to be considered to understand the environmental characteristics that serve to enable or impede PCN processes.

### Framework Basis

The general influence of physical, social, and economic features of the health care delivery system on patient outcomes has been described in Aday's [31] health system evaluation framework and Tarlov and colleagues [32] structural characteristics of care. These generic models delineating factors underlying the three tiers of healthcare (i.e., structure, processes, and outcomes) form the basis of our conceptual framework. To orientate this framework in terms of palliative care, this outline was supplemented by the inclusion of system features from the Ferris and colleagues' [33] Square of Care and Organization model of quality palliative care provision. Developed through a consensus-building process across Canada, this model also takes a S-P-O approach, specifying attributes that should fall under each care level, from Resources and Functions (i.e., structure), to Process, to Common Patient Issues (i.e., outcomes) within the palliative care system. Upon this palliative care system base architecture, the principal constructs of relevance to network functioning are overlaid to create the proposed evaluative conceptual framework. These constructs are: member collaboration, community readiness, and client-centred care.

### Principal Constructs in Framework

There are three key constructs interwoven throughout the framework that are integral to PCN functioning, namely collaborative care, community readiness, and client-centred care. Each of these constructs is described in detail below.

#### Collaborative Care

Inter-professional collaboration where physicians and other health care providers work in partnership to deliver comprehensive and profession appropriate care has gained the attention of policy makers nationally and internationally and has become a priority in most health care reforms [21,34-38]. The desired immediate outcome of PCN formation is the growth of inter-professional provider collaboration [14]. There can be no integration of health care without collaboration; accordingly, consideration of the features of this construct need to be at the core of an examination of these organized networks.

The advantages of a collaborative approach apply extensively to palliative care, where different professional skill sets are required to serve a broad spectrum of patients' needs [11,39]. Meta-analyses have confirmed the benefits of collaborative care, with palliative patients and their families reporting greater satisfaction with health services and better pain and symptom management, as well as improvement in the timeliness of services through expedition of the referral process [2,40-42]. Cost reduction has also been cited as an outcome of collaboration by reducing the amount of time patients spend in acute hospital settings.

Some ambiguity exists in the literature surrounding the term inter-professional health care [21,43]. The prefix 'inter' refers to a partnership where members from different professions work collaboratively towards a common purpose. These partners come together to share ideas, skills, and knowledge to structure a collective action towards the patient's care needs [21,44]. In a multi-disciplinary team, the professional identity and ranking of individual team members usually supersedes team affiliation, whereas in the inter-disciplinary (i.e., inter-professional) team the identity of the team is primary.

Rather than a traditional hierarchy where a physician directs care, in a collaborative approach different team members may assume leadership depending on the patient's needs [6]. Physicians, nurses, and other health care providers have complementary clinical and therapeutic skills, and different perspectives on problems the palliative patient might encounter. With these combined competencies, the inter-professional team is more responsive to the configuration of care delivery required, reducing the complexity of accessing health and social care for patients in need [3]. Synergy is often a defining feature of high functioning collaborative teams, with favourable outcomes possible from collective competences being greater that the sum of the team parts [45,46].

A theoretical model of collaboration should exhibit an understanding of the many elements of the construct and the components influencing the process, at multiple structural levels. Although the health care literature is replete with theoretical frameworks of collaboration, with 29 different models alone reported in a 1995 review [47], no single model lends itself to a comprehensive, practical application and none were designed specifically for palliative care. The majority of published work on the inter-professional collaboration construct relies on conceptual approaches rather than on empirical data [6,48]. This litany of untested frameworks cloud perceptions of exactly which interventions improve collaboration between health care professionals, the influence of determinants on collaboration, and key factors to sustainability [20,49,50]. Identification of the key components of collaboration requires comparison and contrast of the systematic review literature examining this construct, available largely from primary care and chronic care contexts.

#### Community Readiness

The Community Readiness Model is a theory-based approach to ascertaining the favourability of the social and political climate in a given setting to program implementation [51]. This model has been used in international contexts, often to indicate community receptivity to prevention or substance use initiatives [52]. However, community readiness can also be appropriately applied to the planning of community-based health interventions, to predict program sustainability and as a tool for program evaluation.

This model is related to the more commonly cited *organizational readiness *concept [53,54], however, community readiness goes beyond the scope of a single institution in considering multiple organizations, decision makers, and consumers. The manner in which health issues are defined and dealt with is often intertwined with community and cultural norms [51]. Attempting to alter established conventions of health care practice and structure can present a formidable barrier, just as the inertia of traditional care provider roles can impede efforts to increase system integration [55]. In considering community readiness for PCN initiatives, it is important to establish if adequate capacity and support in the general community and between providers and their organizations exists for promoting the network's ideals. System organization, provider education, and policy must all be appropriately aligned to maintain a supportive environment for health professionals practicing inter-disciplinary care.

In many respects, process level factors which imply the state of readiness and shared resolve towards collective action, such as the commitment, attitudes, and goals of both providers and their leaders towards PCN objectives, transcend into the other two principal constructs in the framework: collaborative care and client-centred care. Assessing elements of community readiness, both in system structure and care processes, are an important tenet of predicting the viability of a community palliative care initiative [56]. If a low stage of readiness is indicated, changes proposed by this program are likely to encounter resistance, illuminating the need to first attend to any model elements identified as underdeveloped.

#### Client-centred Care

Client-centred care refers to the provision of care that is respectful of and responsive to individual patient values, needs, and preferences [57]. This includes viewing patients holistically and allowing their unique perspectives to guide care decisions, enabling them to act as a central resource in their own health [58]. Even though the care provider may be the expert clinically, to deliver care that is client-centred requires building a relationship with the patient that facilitates the self-identification of personal goals, to ensure the giving of information and direct care that is appropriate, timely, and pertinent to the client's wishes [59].

Client-centred care has become a key principle of nursing practice in most developed countries and has also been adopted by other health professions [59-62]. This approach has been found to positively impact the satisfaction of both the patient and the provider in primary health care [63]. Likewise, care that is patient focused is vital to the success of patient education and support strategies intended to encourage successful emotional and practical adaptation to advanced chronic illness [58]. Client-centred care has definite applicability to palliative care in the community where support is provided based on the unique circumstances of patients to best sustain their quality of life.

### Conceptual Framework Domains

The components of the framework as presented in Figure 1 are described in the sections that follow. Rationale and empirical support are given for the inclusion of each framework element. This description is organized by system level (S-P-O), with explanation provided for each domain (and sub-domain) within each level.

### System Structure Domains

#### Environment Factors

*Environment factors *are the characteristics of the geographic area or region in which the network system of interest is located. These factors include *population demographics *such as age distribution, income levels, ethnic composition, and cancer mortality rates for the area (see Figure 1). Population density in terms of urban and rural distribution can also have implications for service delivery [56]. Creating a profile of the system environment provides context for comparison to other networks, as well as enabling generalizability of the results to other similar environments.

The capacity for change within the environment largely rests upon the readiness of those living in the planning region's community, including policy makers and consumers. The level of *community awareness *of both the health care issue, e.g., palliative care needs, and the efforts being made to address this problem, as well as the attitude in the community towards the issue, are principal considerations in predicting the success of directed programs [56]. A lack of support in the population for a new initiative can undermine the confidence of providers involved in implementation. A network structure introduced despite community indifference or reservation, which can include opposition by influential organizations, is likely to fail [46,64]. Another prerequisite for a successful PCN is the presence of an adequate pool of nurses, physicians, and allied health care workers with some specialization in palliative care.

#### Network Characteristics

Characteristics of the PCN itself also provide context and indicate system level barriers and facilitators to the achievement of the network's goals and, effectively, optimal outcomes for palliative care patients. Factors to be considered are the PCN's *history*, *evolution*, *structure*, *formal/informal policies and procedures*, and *vision/culture *and whether these aspects promote continued growth of the network and a collaborative environment [33,43,45,48,53,56,65]. The number of health care providers, administrators, and other relevant disciplines in the region holding membership in the PCN (*size of membership*), the *extent of participation *of these members in network functions, and the *promotion of network ideals *internally and throughout the community, all speak to the influence, stability, and perceived value of the PCN. Cooperation between the relevant organizations in the system and their positive regard for the PCN ratifies the commitment of these organizations' employees at a process level. Organizations that have traditionally had substantial control over healthcare resources and service planning may be unwilling to relinquish this power to accept co-dependence, limiting network cohesion [14].

*Policies and procedures *need to reflect clearly delineated obtainable objectives and goals [53,65]. These apply to network development, but also instilling essential features of holistic palliative care. Specifically, this includes policies for consistently offering patient-focused care, home death as a viable option, and expected death planning [66]. A final structural quality is the degree of *power equality *among network members. As previously mentioned, the authority given to each particular professional discipline within the network needs to be equitable to encourage member contribution and support collaborative patient care [48].

#### Economic Factors

Sufficient *resources *and infrastructure are required to make any health program sustainable and adaptive [43,53,56,67]. These assets include cash, financial investments, skilled professionals, equipment, office space, and technology [33]. The availability of designated facility-based programs, namely hospice spaces and palliative care unit beds in tertiary centres, is vital to meeting higher levels of need and providing respite for family caregivers. Liabilities that the PCN carries, such as loans or insurance payments, also need to be considered. The *extent of volunteerism *contributing to either care provider or administrative functions further increases the PCN's capital. *Financial incentives *are the provision of resources tied to the uptake of a specified approach and/or the meeting of set requirements. Incentives aligned with network development provide motivation and legitimacy to this endeavour and therefore need to be noted.

An essential component to palliative care access not found within the constructs considered, that has emerged from review of multiple models of palliative care provision reported in national research documents [68], and as a constituent of integrated care [69], is the system's capacity to offer care 24 hours per day, 7 days per week (*24/7 care*) in each of the relevant professional disciplines. The availability of around-the-clock care is essential to adequately address community palliative care issues such as caregiver fatigue and end-of-life pain and symptom management [66]. Without the availability of 24/7 professional care, crises may arise which result in patient transfer from home to emergency/acute care facilities.

### Process of Care Domains

#### Provider Characteristics

Although inter-professional collaboration and other contributing factors to quality palliative care may be encouraged under the auspices of network structures, these orientations are largely voluntary by nature. Uptake is influenced by the personal characteristics of service providers and administrators in the group [21]. Many of the elements of this domain are contained in Tarlov and colleagues [31] systems of health care model (see Figure 1 for framework). *Provider characteristics *and other process level factors also speak to readiness among the "community" of network members for integrated palliative care [56].

*Beliefs and attitudes *of PCN participants are ideally congruent with those of collaborative client-centred practice in members viewing this endeavour as worthwhile and being motivated towards this end [21,53,65,67,70,71]. *Interpersonal style *refers to professionalism among network members, which ultimately determines the degree to which professionals are able to work together [21,45,65,72,73]. Pertinent interpersonal factors include collegiality, the ability to articulate beliefs and communicate effectively, personal maturity, self-reflection, assertiveness in presenting one's own professions' perspectives with confidence, and willingness to cooperate rather than compete. *Job satisfaction*, bolstered by a favourable work environment, is a strong predictor of provider commitment to their role [45,48,70,71]. Another fundamental characteristic is *specialty training*, representing the professional education and skills of members. The amount of time spent as a palliative care provider and as a network participant are also important considerations [21,45].

#### Extent of Collaboration among Providers

Prior examination of inter-professional teams have shown that collaboration is a complex and dynamic process [21]. While there is a lack of a single definitive model of palliative care inter-professional collaboration to guide a comprehensive evaluation [74], there is some agreement in recent systematic reviews about the key factors within this construct that influence and/or indicate the state of collaborative practice [21,43,45,48,65,67,70,72,75].

Process level factors consistently mentioned in the literature as suggesting the extent of collaboration among providers, are *group commitment, common goals/shared values, perceived interdependence, reciprocity, respect, shared decision-making and problem solving, shared risk/responsibility*, and *trust*. These qualities are also consistent with the prerequisites for supporting client-centred care processes. In fact, some studies interrelate this latter construct with that of collaborative care, such that collaboration leads to desirable client-centred care outcomes [45,73].

Overall *group commitment *to collaboration and to quality care more broadly is one of the most important contributing factors to team functioning and network stability [21,45,48,56,70,71,75]. The *common goals and shared values *sub-domain necessitates members establishing a common language, similar realities and norms, and clear objectives, as well as a team task orientation. The latter implies a collective commitment to excellence in task performance in collaborative client-centred care with minimal conflict [21,45,47,65,67,71,72,75].

*Perceived interdependence *is apparent in team orientation and working relationships among community providers [45,47,56,65,71,72,75]. Having an interdisciplinary team base where team members can work in close physical proximity to one another contributes to interdependence potential. *Reciprocity *refers to the perceived benefits of network involvement for each member weighed against the negative consequences and that there are noticeable returns with increasing collaborative efforts [72,76]. *Mutual respect *implies an appreciation for different professional perspectives and that the contributions of each are valued [21,45,47,48,65,72,75,77]. *Shared decision-making and problem solving *is evidenced in solutions derived through an integration of the expertise of each professional [45,47,71,72]. Furthermore, decisions should be based on general consensus, so that all disciplines feel empowered.

*Shared risk and responsibility *pertains to the accountably for joint initiatives being fairly distributed, with members sharing in the liability for innovations involving risk [21,45,47,65,70,72,77]. Finally, a high level of *mutual trust *within the PCN is quintessential to members working effectively together [21,45,47,48,65,70,72,75,77]. This element infers confidence in others and trusting one's own abilities.

#### Information Transfer

*Communication *pervades all aspects of provider collaboration and patient-centered interactions. Activities that stimulate communication between professionals such as regular formal and informal interdisciplinary forums are crucial to collaboration between individuals and between their organizations, which in turn enhances the quality of palliative care [14]. The sharing of information that is relevant, accurate, transparent, concise, and timely is an essential element for reaching a common understanding across professional boundaries and for constructive negotiations within the network [21,45,47,48,65,71-73,75,77]. The ability and willingness of providers to engage clients in a dialogue unique to their needs and care options captures patient-centred communication from a process level.

*Information systems and materials *are mechanisms to facilitate the exchange of information. Systems include technologies such as pagers, smartphones, electronic health records systems, and multidisciplinary case videoconferencing [43,45,77]. Materials refer to written and visual aids to assist learning, decision making, and uptake of guidelines, network values, and activities. Standardized tools for documenting and transferring information such as an in-home patient chart, also foster effective communication.

Related to informational mechanisms are those for *standardized assessment and monitoring of patient need*. These mechanisms refer to useful clinical tools and assessment instruments and the adoption of these into broader organized approaches within the PCN. Such practices are essential in ensuring that palliative care needs in the community are uniformly identified and that available resources are accessed efficiently [33,68,78].

#### Organization Factors

Organizational factors identified as process determinants of collaborative client-centered care are *care team composition, educational opportunities, incentives to encourage collaboration/client-centred care, leadership, role recognition, standards of practice*, and *process evaluation and feedback*. *Care team composition *refers to the appropriateness of skill mix present in the PCN and having the right people involved both in terms of the expertise they bring to the group but also the influence they have in the community [21,56,65,71,75]. *Educational opportunities *consist of training and workshops, the provision of technical assistance, and venues providing professionals the opportunity to learn from one another [21,71,45,48,79]. *Incentives *to encourage collaborative client-centred care and quality care in general include recognition for innovation and excellence, team building exercises, and time protected for network specific responsibilities. The formalization of these inducements confirms the commitment of executive members to network development [21,71,75]. Research indicates that incentives to encourage collaborative practice are more effective when administered to the team as a whole rather than separately to individuals within the team [71].

*Leadership *is often cited as the single most important factor contributing to collaborative client-centered care. Leaders need to inspire and oversee the other positive process constituents of network functioning and help resolve conflicts that arise [21,46,48,65,71,75,76,79]. Weakness in this role can erode PCN cohesion. *Role recognition *speaks to role clarity in that the contribution of each member is understood, as well as their roles being valued [21,45,48,65,67,71]. *Standards of practice *are benchmarks by which team members can compare current and target values for indices of inter-professional working, client-centred care or other performance qualities [21,45,48,80]. Finally, *process evaluation and feedback *has also been indicated as an essential factor to sustaining network relationships [21,70,75,79,80]. Quality management systems for ongoing evaluation and modification of performance through the review of network activities, functions, and outputs are critical for improving efficiency and enhancing the patient experience.

### Patient Outcome Domains

#### Satisfaction with Domains of Care and Access

The consideration of patient outcomes in the proposed framework does not delve into case complexity, such as functional dependence, which can vary greatly independently of intervention [81]. Rather, this level focuses on the reaction of the palliative care system to needs from patients' points of view. The impact of inter-professional collaboration would be assessed by patients' perceived quality of care and satisfaction with the care they received. A cluster analysis of aspects of palliative care satisfaction in the literature resulted in four dominant sub-domains emerging: *availability of care*, *information giving, physical care *(including *pain and symptom management*), and *psychosocial care *[82]. These factors, along with *management of expected death*, have been identified as the core elements in describing palliative care processes from the care recipient's perspective [21,33,42,70] (see Figure 1).

*Availability of care *simply denotes the perception that services were accessible to those who needed them, when they needed them. The *free flow and accessibility of information *to the patient has also been identified as a central feature of client-centered care [79]. *Physical care *involves medical and practical aspects of care, but also *pain and symptom management *which in this framework has been designated as a separate sub-domain given its importance in palliative care [33]. *Psychosocial care *involves meeting the emotional, psychological, and existential needs of palliative care patients and their families, in helping to alleviate grief, fear, and other psychological and social problems. Finally, *management of expected death *refers to the initiative that providers take to assist family caregivers so that life closure preparations, death pronouncement, certification, and other necessary arrangements occur with little difficulty upon the passing of the palliative individual [33].

#### Perceptions of Client-Centredness of Care

A systematic review by Shaller [79] of nine frameworks for defining client-centered care resulted in the identification of the following core elements of this construct: *appropriate involvement of family and friends *in decision making and information giving; the sense of inter-provider *collaboration and team management*; *education and shared knowledge *in terms of timely and complete information on patient prognosis, progress, and disease process;*respect for patient needs and preferences *in care; and *sensitivity to nonmedical and spiritual dimensions of care*. The end-of-life patient and his/her family perceiving the presence of these factors in interactions with health care providers would imply that care is client centered. Furthermore, this would be particularly evident in patients feeling that care professionals had attempted to build a *rapport *with them, which in turn fostered qualities of interdependence, including trust [58,83].

#### Perceptions of Continuity of Care

An overview by Haggerty, Reid, and McKendry [84] identified the following three types of continuity of care: *relational continuity *(patient seeing usual practitioner); *informational continuity *(communication and knowledge where patient information flows easily between involved care providers); and *management continuity *(coordination of care so that transition between care providers is clear and seamless for the patient). Each of these facets of continuity is another positive outcome facilitated by collaborative relationships between providers, the end result of which should be apparent to patients and their family caregivers [43,45,70]. Accordingly, continuity of care is a phenomenon best measured from the perspective of the patient. Patients' perceiving that efforts had been made by providers to make the care process flow smoothly (*management continuity*) is also an attribute of care that is client-centred [85].

## Discussion

Evaluation and feedback plays a key role in developing organized systems of care [30,75,80]. Evaluation can determine the extent to which program objectives are met, inform policy and planning decisions, and increase community awareness and support for an initiative. Although critical, evaluation can be costly in terms of staff time and funding, and therefore is often deferred in favour of using these resources for providing care [30]. This may explain why the literature is lacking in care delivery research in palliative care [86]. With growing interest in better integrated services for those at end-of-life, there is a global need for whole system research in palliative care that captures the complexity of these initiatives. However, no evaluative framework exists for explicitly examining a network organized system of care in this context.

In this manuscript we have proposed an overarching structure for examining palliative care networks (PCNs) that can be applied to a system evaluation. Findings emerging from such an exercise would be of use to planners, administrators, and advocates of integrated palliative care systems, for the purposes previously mentioned. This paper represents an important initial effort to outline a conceptual map of the system structure, process of care, and patient outcome (S-P-O) domains for organizing systems of care for those in the palliative phase of life who are residing in the community. Research evidence was used to substantiate the choice of model factors. Our focus has been on inter-professional collaborative palliative practice, client-centred care, and community readiness constructs; taking into account contextual factors to capture the unique features of the system environment. Many of the elements derived from these constructs overlap, indicating their interdependence.

This model does not incorporate specific palliative care practices, as have been proposed by intervention guidelines such as the Liverpool Care Pathway [87]. Nor have we considered individual patient and family characteristics, which often play a decisive role in patient and caregiver outcomes [88], but yet likely reflect intrinsic attributes and dispositions that are less modifiable through formal support interventions [89]. Instead our framework is built around features of importance to PCN functioning, with provider and patient contexts. Many of the factors contained herein could also be suitably applied to the examination of integrated community systems for the management of other diseases.

In terms of application, the proposed framework assumes the employment of a mixed-methods research plan. This implies using multiple sources of data to consider the different perspectives and S-P-O levels within the PCN system. Mixed-method research is commonly advocated to provide for a broader range of questions, leading to a more complete understanding of the phenomena of study - in line with a systems approach [90,91]. Compared to single method studies, stronger evidence is possible through using mixed-methods in the convergence and corroboration of findings, with the results having greater generalizability. A case study methodology is one type of mixed-methods design that complements the proposed framework [92,93].

At the structure level, data would be obtained from PCN administrators and document review (e.g., meeting minutes, presentations, etc.). Data collection at the process level would need to include members of the PCN providing care, these being specialist nurses, primary care and palliative care physicians, pharmacists, therapists, and social workers [6,94-96]. As for patient outcomes, obtaining responses from palliative individuals can be challenging [81,97,98]. As such, it may be prudent to collect data from the primary family caregiver rather than the patient directly to gain insight into care outcomes in the community [99]. A factor matrix based on the questionnaire items developed from the conceptual framework should be created to ensure complete coverage of desired elements and to assist in analysis for mapping individual factors relative to one another. Visually depicting the data in an organized array is a useful step in progressing theme formation and in discerning relationships between the structure, provider, and patient levels of the system.

While comprehensiveness is a strength of this proposed conceptual framework, it can also be a limitation in the breadth being possibly too extensive for the practical evaluative needs of a PCN. In translating the framework into an evaluation research plan, attempting to consider all the dimensions and numerous factors at once can make operationalization a challenge, particularly if time and resources are limited. Evaluators, especially health care providers taking on this role in addition to their clinical responsibilities, should avoid making data collection too burdensome [80]. Whether it is a one-time snapshot of the network or the implementation of an ongoing surveillance mechanism, it may be advisable to start small to avoid the process becoming unmanageable and subsequently being abandoned entirely. Depending on the objectives of the inquiry, the size of the program, and the resident experience present, researchers may prioritize select elements to be included as sentinel indicators, to focus measurement efforts.

One approach to a more pragmatic examination is to limit the inquiry to issues at the patient level and then trace problems that emerge back to the processes of care to isolate and attend to contributing factors. Alternatively, a selection of factors at the process level could be assessed by using an existing validated tool that captures some of the domain(s) of interest. For example, the general state of inter-professional collaboration could be determined using an established instrument such as the Partnership Self-Assessment Tool (PSAT) [46], which is regarded as one of the better instruments to measure this construct [72] and includes a reporting module for interpreting and disseminating the results. Further testing of the proposed model may reveal a set of core indicators in network functioning and outcomes that lend themselves to the creation of a condensed version of the model, for guiding a basic evaluation of a PCN.

A network approach to service provision does not necessarily assure the best care for patients, particularly if health care funding streams continue to be competitive and encourage provider organizations to be territorial. A system driven by a single authority could potentially be more efficient and produce better outcomes than a cooperative arrangement, by dissolving organizational boundaries. Still, given the diversity of providers and organizations providing care to end-of-life patients and their families in many communities and the variable needs of this patient population, attempting to integrate existing programs seems a more feasible solution. The provision of palliative care that is client focused needs to be the grounding objective in service integration to help refocus some of the territoriality that arises as individual organizations try to protect their own interests [14].

## Conclusions

It is important to remember that system integration, however advantageous, takes a long time to achieve [46,100], requiring resources and the participation of the full range of palliative care providers, from hospitals to independent practitioners. The conceptual framework proposed contains a multiplicity of key factors to palliative care system functioning. This is a first step to guide evaluation to inform the development of appropriate strategies to further promote collaboration within the PCN and, ultimately, optimal palliative care that meets patients' needs and expectations.

## Competing interests

The authors declare that they have no competing interests.

## Authors' contributions

DB performed the search and analysis of background materials. DB and KB drafted the manuscript. PK, JP, and AT reviewed the manuscript critically for intellectual content. All authors read and approved the final manuscript.

## Pre-publication history

The pre-publication history for this paper can be accessed here:

http://www.biomedcentral.com/1472-684X/9/8/prepub
